# Japanese Encephalitis in Small-Scale Pig Farming in Rural Cambodia: Pig Seroprevalence and Farmer Awareness

**DOI:** 10.3390/pathogens10050578

**Published:** 2021-05-10

**Authors:** Ellinor Henriksson, Rebecca Söderberg, Gunilla Ström Hallenberg, Kang Kroesna, Sokong Ly, Borin Sear, Fred Unger, Sothyra Tum, Hung Nguyen-Viet, Johanna F. Lindahl

**Affiliations:** 1 Department of Clinical Sciences, Swedish University of Agricultural Sciences, 750 07 Uppsala, Sweden; ellinorhenriksson@hotmail.com (E.H.); rebecca.soderberg@telia.com (R.S.); gunillastrom1@gmail.com (G.S.H.); 2 Public Health Agency Sweden, 171 65 Stockholm, Sweden; 3 Faculty of Veterinary Medicine, Royal University of Agriculture, Phnom Penh 12201, Cambodia; kkroesna@rua.edu.kh (K.K.); leesokong@gmail.com (S.L.); borin_sear2007@yahoo.com (B.S.); 4 Animal and Human Health Program, International Livestock Research Institute, Hanoi 100 000, Vietnam; F.Unger@cgiar.org (F.U.); H.Nguyen@cgiar.org (H.N.-V.); 5 National Animal Health and Production Research Institute, General Directorate of Animal Health and Production, Phnom Penh 12350, Cambodia; sothyratum@gmail.com; 6 Department of Medical Biochemistry and Microbiology, Uppsala University, 751 23 Uppsala, Sweden

**Keywords:** zoonosis, vector-borne disease, arbovirus, neglected disease, pig farming, Southeast Asia

## Abstract

Japanese encephalitis (JE) is endemic in Cambodia, but circulation of JE virus (JEV) among domestic pigs has previously only been studied in the southern part of the country. The main purpose of this study was to determine the seroprevalence of JEV antibodies in smallholder pigs held in rural areas of Kampong Thom, Preah Vihear, Ratanakiri, and Stung Treng provinces, northeastern Cambodia. Another purpose was to identify possible associations between serologic status and other factors, such as reproductive disorders, and to investigate the farmers’ knowledge of mosquito-borne diseases and use of preventive measures. In October 2019, 139 households were visited throughout the study area, and 242 pigs were sampled for blood. The sera were analysed with ELISA for JEV antibodies. Household representatives were interviewed, and data were recorded for each sampled pig. The apparent seroprevalence was 89.1% in pigs between 3 and 6 months of age, and 100% in pigs over 6 months of age. In total, 93.0% of the pigs tested positive. Province appeared to be the only factor significantly associated with serologic status (*p* < 0.001). Almost all (97.8%) respondents knew that mosquitos could transmit diseases, and 70.5% had heard of JE. However, only one respondent knew that JEV is transmitted to people through mosquito bites. Very few respondents knew that pigs can become infected with JEV, and no one knew that mosquitos transmit the virus. All families used some sort of mosquito protection for themselves, but only 15.1% protected their pigs from mosquito bites. The children were vaccinated against JE in 93 households, while adults only were vaccinated in eight households. The results suggest that JEV transmission is intense in northeastern Cambodia, and that people’s knowledge about the transmission route of JEV and the role of pigs in the transmission cycle is low. Fortunately, people are well aware of mosquito-borne diseases in general and use mosquito protection, and many children are vaccinated against JE. Nonetheless, it is important that national vaccination is continued, and that people—especially in rural areas where pigs are commonly kept—are educated on the ecology and transmission of JEV.

## 1. Introduction

Japanese encephalitis (JE) is a serious neurologic disease and a major health concern in many Asian countries, including Cambodia. It is caused by Japanese encephalitis virus (JEV), a mosquito-borne flavivirus that infects several vertebrate species, although mainly pigs, horses, and humans develop clinical symptoms [1]. The major mosquito vectors prefer to breed in rice fields, and pigs are considered important amplifying hosts [2]. Infection in humans may cause encephalitis and death, or neuropsychiatric sequela among survivors [3]. In endemic areas, such as Cambodia, JE is considered a childhood disease, as most adults have acquired active immunity through repeated exposure to JEV [1]. The disease is generally considered rural, and proximity to rice fields and pigs are associated with increased risk of transmission.

In 2018, Cambodia had a population of 16.2 million people, of which 12.4 million resided in rural areas [4]. Rice cultivation and pig farming are common practices, especially in the countryside [5]. Many families are smallholders that keep their pigs close to their homes, with less than 20% of the farms counting as large scale [5]. Therefore, a major part of the population is at risk of infection. Cases of clinical JE occur year-round in children under 15 years of age. In 2007, the incidence was estimated to be 11.1 per 100,000 children [6]. The circulation of JEV is high, as most pigs seroconvert before 6 months of age [7]. Apart from being a threat to human health, JE manifests in reproductive disorders in infected sows and boars [8] and may have a negative impact on families’ livelihoods, as pig farming generates both income and food.

The main purpose of this study was to determine the seroprevalence of JEV antibodies in smallholder pigs in rural parts of Kampong Thom, Preah Vihear, Ratanakiri, and Stung Treng provinces, northeastern Cambodia. Although JE is considered endemic, the circulation of JEV among pigs has previously only been studied in the southern part of the country. Another purpose was to identify possible associations between serologic status and animal characteristics, pig management, and reproductive disorders, and to investigate the smallholders’ knowledge of mosquito-borne diseases and use of preventive measures. In 2016, vaccination against JE was incorporated into the national childhood immunization program [9]. This study explores how well this program has succeeded in reaching children in these areas.

## 2. Results

In total, 139 households were included in the study, of which 39 were located in Kampong Thom, 34 in Preah Vihear, 35 in Ratanakiri, and 31 in Stung Treng. Of the respondents answering the questionnaire, 75.4% were female and 24.6% were male. The mean age was 41 years, ranging from 18 to 74. Most had finished primary school (42.6%), but 34.6% had no education, and only one respondent had attained a college or university degree. The share of uneducated respondents was highest in Kampong Thom (48.7%) and lowest in Stung Treng (12.9%). The mean number of pigs kept by the households was 4.2, ranging from 1 to 20. No pigs were kept in buildings that provided protection against mosquitoes.

### 2.1. Seroprevalence

In total, 242 pigs were sampled, of which 63 came from Kampong Thom, 51 from Preah Vihear, 63 from Ratanakiri, and 65 from Stung Treng. The sampled pigs were between 3 months and 5 years of age, with the mean age being 9 months. The majority (60.9%) were female. In Preah Vihear and Stung Treng, over 90% of the sampled pigs were indigenous breeds compared with Kampong Thom and Ratanakiri, where around half of the pigs were indigenous breeds. The rest of the sampled pigs were mainly exotic breeds and very few were crossbreeds.

**Figure 1:**
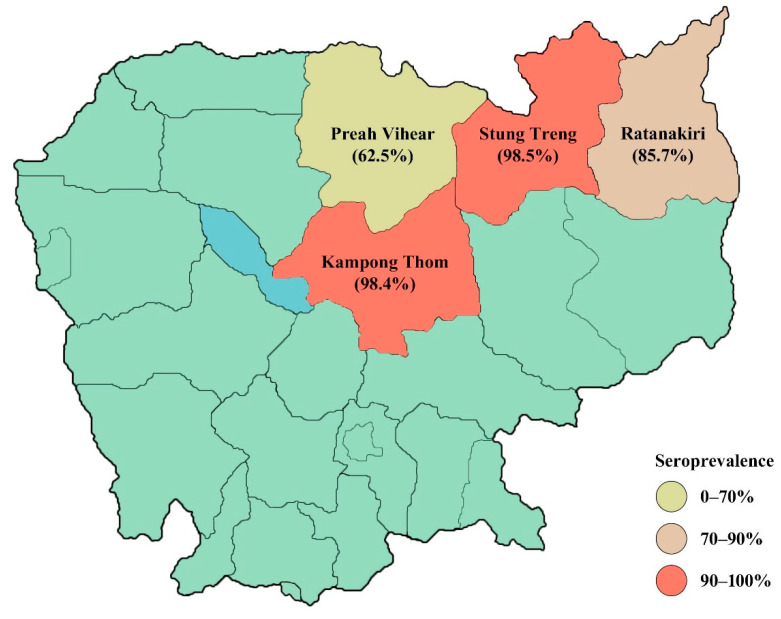
Map of Cambodia, the studied provinces, and the seroprevalence of JEV antibodies among pigs from each province.

Of the collected samples, most generated valid test results. However, in Preah Vihear, only eight samples could be interpreted since the OD values of the positive and negative test controls were similar on one of the ELISA plates. In total, 185 (93.0%) of the 199 samples that generated valid test results were positive (Table 1). All pigs over 6 months of age had detectable JEV antibodies compared with 89.1% of pigs under 6 months of age. However, age was not significantly correlated with serologic status (*p* = 0.087). There was a significant difference in seroprevalence between the provinces (*p* < 0.001). The seroprevalence was highest in Stung Treng (98.5%) and lowest in Preah Vihear (62.5%) (Table 1 and Figure 1). The mean age of seropositive pigs was 8.6 months (95% confidence interval (CI) 7.2–10.1) compared with 4 months in seronegative pigs (95% CI 3.5–4.5). Kampong Thom had the highest share of tested pigs over 6 months of age, while Stung Treng had the lowest (Table 2). All pigs that were crossbreeds tested positive, as did most pigs of commercial (96.8%) and indigenous (91.0%) breeds. Type of breed was not significantly associated with seropositivity (*p* = 0.384).

Households with at least one seropositive pig were considered “seropositive households.” Of the households that let their pigs roam freely during the dry season, the rainy season, or both seasons, 92.3% had at least one pig that were seropositive compared with 94.1% of the households that always kept their pigs confined or tethered. Around 91% of households that were in sight of rice fields were seropositive compared with 97.4% of other households. Neither housing system (*p* = 0.668) nor proximity to rice fields (*p* = 0.213) were significantly associated with serologic status of households.

The multivariable logistic model included the variables identified as associated with pig level seropositivity at *p* < 0.2. Results showed that increasing age and the province Stung Treng increased the odds ratio compared to Ratanakiri, while Preah Vihear had a lower odds ratio (Table 3).

### 2.2. Reproductive Disorders

There were no reports of abortions in any of the sampled sows during the past year. Only one sow was known to have had both stillborn and mummified fetuses, two to have had stillborn fetuses, and one to have had mummified fetuses. Test results were only valid for one of these sows, which was positive for JEV. However, when respondents were asked if any of their sows had aborted during the past year, 24.4% answered ‘Yes.’ Stillborn or mummified fetuses were observed in 18.5% and 10.1% of households, while 9.3% and 11.2% had had weak or shaking piglets. There was no significant association between reported abortions and serologic status of households (*p* = 0.336, Table 4). However, none of the seronegative households had experienced any abortions. Among the seropositive households, 22.9% had experienced abortions. Furthermore, no seronegative households had observed any stillborn or mummified fetuses, whereas 31.5% of the seropositive households had observed either (Table 5). However, these differences were not significant (*p* = 0.519). Weak or shaking piglets had not been observed in seronegative households but were reported by 13.0% of the seropositive households. Similar to abortions, stillbirths, and mummified fetuses, there was no significant correlation between either weak (*p* = 1.000) or shaking (*p* = 1.000) piglets and serologic status of households despite the absence of such piglets in seronegative households.

### 2.3. Disease Knowledge and Prevention

Of all 139 respondents, 98 (70.5%) reported to have heard of Japanese encephalitis (Table 6), whereas 21 (21.4%) claimed that they knew what it is but only six could deliver an explanation by describing clinical signs, such as fever, headache, diarrhea, and salivation. Of those who reported to have heard of JE, 93 (94.9%) knew that humans can become infected, but only seven (7.1%) knew that pigs are susceptible. Only six (6.5%) of those who knew that people could become infected claimed that they knew how. Three delivered an explanation, although only one correctly explained it as being transmitted by mosquitos. Of the respondents who knew that pigs could become infected, only one claimed to know how but gave no explanation. Four respondents had somebody in their family who had had JE, and four knew people outside of the family who had had it. One respondent reported that there had been one JE-caused death in the village. There were no significant relationships between disease knowledge (respondents who had heard of JE) and sex (*p* = 0.661) or education level (*p* = 0.316) of the respondent.

**Figure 2:**
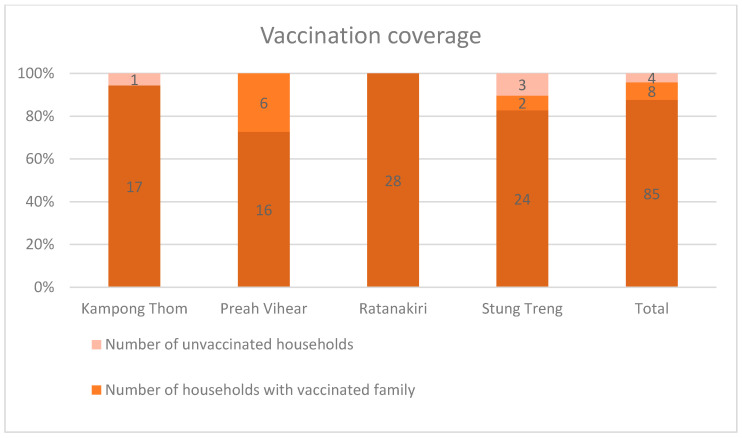
Vaccination coverage in the studied provinces (numbers are indicated as numerals in bars).

When respondents that had heard of JE were asked if their family was vaccinated, all replied but one. Of these, eight (8.2%) reported that the whole family was vaccinated against JE, whereas in 85 (87.6%) of the households, only the children were vaccinated against JE. In total, 93 (95.9%) households had vaccinated children. In all households (84) that answered the question of who had paid for the vaccination, respondents reported that it had been funded by the government. Households that were not vaccinated were asked to explain why not. Of these, one respondent explained that they did not know that people can become infected with JEV. Another respondent answered that they were afraid of vaccine side effects. There was no significant association between vaccinated households (any household with vaccinated family members) and province (*p* = 0.149). However, there was a significant difference between the provinces (*p* = 0.002) for households where all family members were vaccinated. In Preah Vihear and Stung Treng, the whole family was vaccinated in 27.3% and 6.9% of the households, and in Kampong Thom and Ratanakiri, there were no such households (Figure 2).

Of 137 respondents, 134 (97.8%) reported to have heard of diseases being transmitted to people through mosquito bites. All 139 households used some sort of mosquito protection: 96.4% used bed nets, 56.8% used insect repellents, and 5.8% used covering clothes. In total, 21 (15.1%) households protected their pigs in some way. Of these households, 47.6% used insect repellents, 14.3% used smoke, one (4.8%) used mosquito nets, and one used light. The remaining households did not specify how they protected their pigs. There was no significant association between protection of pigs from mosquito bites and serologic status of households (*p* = 1.000).

## 3. Discussion

The apparent prevalence of JEV antibodies observed in this study was very high, as all pigs over 6 months of age and 89.1% of pigs from 3 to 6 months of age tested positive with the ELISA. The high seroprevalence, as well as the slight difference between these age groups, was in correspondence with results of other studies on JEV infection in pigs in Cambodia. Duong et al. [7] found that 95.2% of pigs between 6 months and 1 year of age were positive compared with 87.3% of pigs between 4 and 6 months of age. Cappelle et al. [10] and di Francesco et al. [11] found that more than 98% of the monitored pigs seroconverted before 6 months of age. The reason for the lower prevalence in younger pigs is most likely explained by the weaning of maternal antibodies around 3 months of age followed by the varying, although relatively high infection rate in susceptible pigs, as observed by Cappelle et al. [10] and di Francesco et al. [11]. A recent study in Vietnam found that the seroprevalence among pigs between 4 and 5 months of age was even lower; 53–74% [12]. However, the seroprevalence among adult pigs in Vietnam has been found to be 100% [13]. The ELISA test used in the present study does not distinguish between actively produced and passively acquired JEV antibodies. Therefore, it cannot be assumed that all positive pigs under 6 months of age have been infected with JEV, as some may be positive because they still are protected by maternal antibodies. As reported by Scherer et al. [14], most pigs lose their passive immunity between 4 and 6 months of age, but for some, maternal antibodies are still detectable at 7 months of age.

There was a significant difference in seroprevalence between the provinces included in this study. However, for Preah Vihear, where the prevalence (62.5%) was remarkably lower than in the other provinces, 43 of the samples could not be interpreted and were thus excluded from the data analyses. Only eight samples remained, of which five were positive. The low number of included samples is therefore not representative of the whole province. However, Duong et al. [7] found that the prevalence varied between the eight Cambodian provinces included in their study. In three of the studied provinces, all pigs between 2 months and 1 year of age were positive compared with 52.5% in Kampong Cham and 60% in Takeo. Because of the large sample size and the big difference between provinces, a lower prevalence in Preah Vihear cannot be ruled out.

There were no significant associations between reproductive disorders and serologic status of households. However, abortions, stillbirths, mummified fetuses, and weak or shaking piglets had not been observed in any of the seronegative households during the past year. Reproductive disorders had not been observed in most of the seropositive households either, but the total absence in all seronegative households is still an interesting finding. However, as all pigs over 6 months of age were seropositive, the presence of seronegative pigs in seronegative households is not an indication of the serological status in adult, sexually mature pigs. Moreover, the fact that pigs are seropositive does not provide any information about when they became infected. Thus, it is possible that pigs do not become infected with JEV until after they reach sexual maturity. If infection occurs before 60 to 70 days of gestation in gilts or sows, then the virus can cause reproductive disorders [8]. However, given the endemic situation in the country, most pigs probably become infected before reaching sexual maturity, and it is unlikely to find an association between serologic status and reproductive performance [15]. It is also possible that the study was underpowered to detect small differences with this high prevalence. The households included in the present study were asked if they had observed any reproductive disorders during the past year, and their responses may have been influenced by recall bias given the long time period. The ongoing outbreak of African Swine Fever in Cambodia and many other Asian countries in 2019 may also have affected farmer responses, since the provincial official veterinarian was always present during the interviews, and farmers might have been cautious to mention if their pigs had showed any signs of disease.

Almost all respondents had heard of mosquito-borne diseases, and over two-thirds had heard of JE. However, only one person knew that people become infected with JEV through mosquito bites. This indicates that there is a scope to improve knowledge about the transmission of JEV, as well as other vector-borne diseases in these rural provinces. Although the knowledge of JEV transmission appeared to be low, all households used some type of mosquito protection. Almost all households used bed nets and more than half used insect repellent, although the frequency of application is unknown. Because almost all respondents were aware of that mosquito bites can cause disease, they were likely motivated to protect themselves, although practical inconvenience or high costs might influence the frequency of application.

Only one household used mosquito nets to protect their pigs, while ten used insect repellents and three used smoke. Households that did not protect their pigs were not asked why, although the lack of knowledge about that pigs can become infected with JEV through mosquito bites, as was found in this study, might be an explanation. Because JEV infection is usually subclinical in pigs [8], it is understandable if pig farmers are not aware of the infection risk. The lack of knowledge regarding the JEV infection route to both pigs and humans indicates that there is a need for more education on JEV and strategies to suppress virus circulation and avoid infection. This may be achieved through information campaigns and/or elementary school education. The effects of mosquito protection for pigs would need to be evaluated in the ecological setting it is intended for, since there are many factors that influence the intensity of JEV transmission, such as precipitation [16], mosquito breeding grounds [17] and amplifying birds [2], and there may be other preventive measures that are more cost-effective. In this study, there was no observed difference in serologic status between the households that protected their pigs and the other households regarding serologic status.

Households that had heard of JE (which represented two-thirds of all households) were asked whether they were vaccinated against JE or not. The majority of these had vaccinated children, and more than 90% of the vaccinations were funded by the government. There was no significant difference between the provinces regarding the prevalence of households with vaccinated children, and the vaccination coverage appeared to be high for children in all provinces. The incorporation of JEV into the national childhood immunization program in 2016 [9] has likely been successful in reaching children of rural families in these areas.

One proposed strategy to reduce the transmission of JEV is to vaccinate pigs. However, there are many reasons for why this strategy would not be applicable to the Cambodian context. First, which is a general liability, the turnover rate in pig production is high, and vaccination would need to be performed frequently and would require high costs in terms of both management and investment [8]. Second, because most adult sows in Cambodia are likely to be immune to JEV, as observed in this study and by Duong et al. [7], piglets acquire passive immunity through colostrum intake. The presence of maternal antibodies could interfere with vaccination, making it ineffective [8]. Also, there is a relatively short window between the waning of maternal antibodies at 2 to 3 months of age and the development of active immunity through natural infection with JEV, as most pigs in Cambodia start producing antibodies in response to infection before 6 months of age [10]. Third, vaccination of pigs does not eliminate the risk of human infection, since JEV can also be transmitted from birds [2,18]. Many households in Cambodia keep chickens or ducks [11,19], which have potential to infect mosquito vectors with JEV [20]. Moreover, wild aquatic birds such as herons and egrets—the natural maintenance reservoir for JEV [2]—are present in Cambodia.

Another proposed control strategy is to reduce the number of mosquito vectors by reducing the number of larvae in rice fields. Keiser et al. [17] compared chemical and biological intervention strategies and concluded that one of the most sustainable and worthwhile strategies is alternate wet and dry irrigation (AWDI). However, this strategy can only be performed in settings where irrigation water can be managed. The irrigation infrastructure in Cambodia has grown gradually since the 1990s but remains significantly underdeveloped [21]. In 2010, approximately 24% of the rice land was estimated to be irrigated. Most rice fields are rain fed, with or without supplementary irrigation. Drainage systems are poorly developed, and floods are frequent during the rainy season. Because of limited irrigation and drainage possibilities, AWDI may not be very feasible in Cambodia. If the water supply and drainage of rice fields could be controlled, then mosquito vectors could potentially be reduced.

A possible source of false test results in this study is the unknown specificity of the ELISA test. It is designed to detect antibodies against the envelope protein of West Nile virus, but cross-reacts with antibodies against other flaviviruses, which exhibit the same type of protein. The test has previously been successfully used to detect JEV antibodies in sera from pigs in Vietnam [22]. In that study, 105 of 108 ELISA-positive samples were confirmed to be truly positive for JEV with virus neutralization test, which is a highly specific serological assay used to distinguish between antibodies against different flaviviruses. Except for JEV, the only flaviviruses present in Cambodia that can infect and induce antibody production in pigs are the Dengue and Zika viruses [8,23,24,25]. However, infection with these viruses has only been observed in experimentally infected pigs [8], and the circulation of Zika virus is low in Cambodia [23]. Thus, it is unlikely that the ELISA would have cross-reacted with antibodies against any other flaviviruses than JEV. Because there are no known sensitivity or specificity values, the predictive value of a positive test (PVPT) could not be calculated. However, since the PVPT correlates with prevalence and the apparent prevalence was very high, most of the positive rest results are likely to be true positive.

## 4. Materials and Methods

### 4.1. Study Area and Selection of Households

This cross-sectional study was conducted in October 2019 in four rural provinces in Cambodia: Kampong Thom, Preah Vihear, Ratanakiri, and Stung Treng. These provinces were purposely selected as they were considered to have the highest level of extensively kept pigs in the country by the national authority the National Animal Health and Production Research Institute (NAHPRI, Phnom Penh, Cambodia). In each province, three to four pig-abundant, geographically dispersed districts were selected by the provincial veterinarians. Within each district, smallholder farmers keeping up to ten pigs older than 3 months were purposively selected with the assistance of the village head. All farmers were informed about the study and asked for their consent to participate.

### 4.2. Data Collection

In each participating household, one adult person (over 15 years old) responsible for the pigs was interviewed about their pig management, occurrence of reproductive disorders among the pigs, knowledge of JE and mosquito-borne diseases in general, and disease prevention using a structured questionnaire. The questionnaire (Supplementary Material 1) was developed in English after expert consultations with NAHPRI, the Royal University of Agriculture (RUA), and the International Livestock Research Institute (ILRI). The questionnaire was then translated to Khmer by a veterinary student from the Royal University of Agriculture, who conducted the interviews together with 2 fellow veterinary students. The questionnaire was tested in the initial interviews, but no changes were deemed necessary. No further validation was done.

In each household, blood samples were collected from one to three pigs over 3 months of age (to avoid interference of maternal antibodies). When possible, pigs of various ages were selected, including at least one sow. Pregnant sows and pigs that showed any signs of illness were excluded to avoid stressing the animals. Blood was sampled from the jugular or ear vein of the pigs into Vacuette serum tubes, which were individually labelled and kept in a cooling box. The samples were centrifuged within 24 h, and sera were transferred to labelled cryotubes and stored in a freezer (−18 °C). During transportation to the laboratory in Phnom Penh, the samples were kept in a cooling box on ice packs. For each sampled pig, a form was filled out (Supplementary Material 2), including information about sex, age, breed, and reproductive disorders.

### 4.3. Serological Analysis

Samples were analyzed in the laboratory of NAHPRI in Phnom Penh. Presence of JEV antibodies in sera was tested through competitive enzyme-linked immunosorbent assay (ELISA). Commercial ID Screen West Nile Competition Multi-species kits (Innovative Diagnostics, Grabels, France), designed to detect antibodies against the envelope protein of West Nile virus (WNV), were used. The same ELISA kit was used in a previous study in Vietnam, which confirmed that 97.2% of the test seropositive were in fact JEV seropositive using virus neutralization test (VNT) [22]. In this study, only the ELISA was used. The test was conducted and interpreted according to the manufacturer’s instruction defining seropositivity in the test as JEV, as WNV is not supposed to be present in the region. All tests were validated against positive and negative test controls according to the manufacturer’s instruction. Whereas the ELISA is highly sensitive [26], it is less specific than VNT. Further, since ELISA is a multispecies test, it has not been validated for pigs, and has been validated less so for JEV antibodies. Therefore, it is not possible to give the assay a specificity or sensitivity value.

### 4.4. Statistical Analyses

Descriptive and statistical analyses of data collected through the questionnaire, blood sample form, and serological analysis of samples were conducted in Microsoft Excel and STATA 14.2 (STATACorp Ltd., College Station, TX, USA). The significance of potential associations between serologic status in individual pigs or of households and other factors, including age and reproductive disorders, was determined through *t*-test (age) and Fisher’s Exact test (age and other factors). Seropositive households were defined as households keeping one or more seropositive pigs. Associations between disease knowledge and sex or education of the respondent, and between vaccinated households and province, were also investigated, using Fisher’s Exact Test. No corrections were performed to account for multiple comparisons, and a *p*-value under 0.05 was considered significant. Variables with a univariable association of *p* < 0.2 with pig level seropositivity were included in a multivariable logistic regression using STATA command logit.

### 4.5. Ethics Statement

All participating farmers gave informed consent. The project was part of a larger project with ethical approval received from the National Ethical Committee of Cambodia, coded 300NECHR, dated 26 December 2017, and was approved by the National Animal Health and Production Research Institute, General Directorate of Animal Health and Production.

## 5. Conclusions

The results of this study confirm that JEV is indeed endemic in Cambodia, and that most pigs become infected before reaching sexual maturity. No risk factors for infection apart from province could be identified because of the high seroprevalence in the study population. Almost all respondents had heard of mosquito-borne diseases and protected themselves from mosquito bites. Although most respondents had heard of JE, only one could correctly explain how people become infected. No one knew that pigs can become infected with JEV through mosquito bites, and less than one in five households used mosquito protection for their pigs. The low knowledge of the role of pigs and mosquitos in the amplification and transmission of the virus indicates that more education on the ecology of JEV is needed, especially targeting people that live in rural areas in proximity of pigs. The vaccination coverage in children of rural families in Kampong Thom, Preah Vihear, Ratanakiri, and Stung Treng appears to be satisfactory. However, national vaccination of children should be continued to prevent cases of JE in Cambodia, where JEV transmission is highly intense, as observed in this study.

## Figures and Tables

**Table 1:** Seroprevalence of JEV antibodies among pigs of different age categories from four provinces in Cambodia.

Age Category	Seropositive Pigs % (*n*)	Total Number of Pigs
3–5 months	89.1 (114)	128
6–11 months	100 (31)	31
≥12 months	100 (38)	38
All	92.9 (183)	197 *
**Province**		
Kampong Thom	98.4 (62)	63
Preah Vihear	62.5 (5)	8
Ratanakiri	85.7 (54)	63
Stung Treng	98.5 (64)	65
All	93.0 (185)	199

* The age was not recorded for two of the seropositive pigs, hence only 197 pigs are included in the age analysis.

**Table 2:** Distribution of tested pigs of different age categories between the studied provinces.

Province	3–5 Months % (*n*)	6–11 Months % (*n*)	≥12 Months % (*n*)	Total Number of Pigs
Kampong Thom	47.6 (30)	17.5 (11)	34.9 (22)	63
Preah Vihear	50.0 (3)	0.0 (0)	50.0 (3)	6
Ratanakiri	71.4 (45)	15.9 (10)	12.7 (8)	63
Stung Treng	76.9 (50)	15.4 (10)	7.7 (5)	65
All	65.0 (128)	15.7 (31)	19.3 (38)	197

**Table 3:** Results from multivariable logistic regression model for seropositivity for Japanese encephalitis.

Risk Factor	Odds Ratio	95% Confidence Interval	*p*-Value
Age (month)	1.7	1.0–2.8	0.040
Stung Treng	11.2	1.4–92.3	0.025
Kampong Thom	7.6	0.9–63.4	0.061
Preah Vihear	0.01	0.0–0.7	0.031
Ratanakiri	ref		
Constant	0.6	0.08–4.7	0.6

**Table 4:** Correlation between serologic status of households and occurrence of abortions.

	Abortions		
JE Household	No	Yes	Total
Negative	6 (100%)	0 (0%)	6
Positive	74 (77.1%)	22 (22.9%)	96
All	80 (78.4%)	22 (21.6%)	102

**Table 5:** Correlation between serologic status of households and occurrence of stillbirths or mummified fetuses.

	Stillbirths or Mummified Fetuses			
JE Household	No	Yes, Stillbirths	Yes, Mummified Fetuses	Total
Negative	5 (100%)	0 (0%)	0 (0%)	5
Positive	61 (68.5%)	22 (24.7%)	6 (6.7%)	89
All	66 (70.2%)	22 (23.4%)	6 (6.4%)	94

**Table 6:** Knowledge of JE among respondents.

Question	Answer	Respondents % (*n*)	Total Number of Respondents
Heard of JE?	Yes	70.5 (98)	139
	No	29.5 (41)	
Susceptible species?	Humans	94.9 (93)	98
	Pigs	7.1 (7)	
Infection route in people?	Mosquitos	1.1 (1)	93
Infection route in pigs?	Mosquitos	0 (0)	7

## Data Availability

The supporting data will be made available by the corresponding author upon request.

## Supplementary Materials

The following are available online at https://www.mdpi.com/article/10.3390/pathogens10050578/s1, File 1: Questionnaire, File 2: Blood sample form.
