# Supplementary material for: Japanese Encephalitis in Small-Scale Pig Farming in Rural Cambodia: Pig Seroprevalence and Farmer Awareness
Source: Pathogens. 2021 May 10;10(5):578. doi: 10.3390/pathogens10050578 (PMC8150308; doi:10.3390/pathogens10050578)
Supplement: Supplementary file 1 [file pathogens-10-00578-s001.zip › pathogens-1144450-supplementary.pdf]

Supplementary material 1- Questionnaire

○ Used for questions with only one possible answer

□ Used for questions with more than one possible answer

Farm no: ……….

No	A. Household demographics		
**A1**	Geographic location	Province: ....<br>District: ....<br>Commune: ....<br>Village:	
**A2**	Sex of respondent	Female ○<br>Male ○	
**A3**	Age of respondent	.... years	
**A4**	Highest level of education of respondent	College/University ○<br>Upper secondary school ○<br>Lower secondary school ○<br>Primary school ○<br>No education ○	
**A5**	Number of people living in the household	Adults (15-60 years): ……….<br>Children (< 15 years): ……….<br>Elderly (> 60 years): ……….	
No	***C. Farm details***		
**C1**	Number of pigs (at time of visit)	Piglets (< 1 month): ……….<br>Growers (1-3 months): ……….<br>Fatteners (> 3 months): ……….<br>Breeding sows: ……….<br>Breeding boars: ……….	
**C2**	Pig breed(s) on the farm	Indigenous pigs □<br>Exotic pigs □	
**C3**	Housing system for the pigs	Tethered □	

		Confined in pens ☐<br>Partly confined ☐<br>Free roaming ☐	**Go to C6**
**C4**	Which group(s) of pigs are tethered/confined?	Piglets (< 1 month) ☐<br>Growers (1-3 months) ☐<br>Fatteners (> 3 months) ☐<br>Breeding sows ☐<br>Breeding boars ☐	
**C5**	If partly confined, during what part of the day are the pigs kept confined?	Day ☐<br>Dusk ☐<br>Night ☐<br>Other: ………. ☐	
**C6**	If partly confined/free roaming, during which season do the pigs roam free?	Dry season ○<br>Rainy season ○<br>Both ○	
No	***D. Disease knowledge and vaccination***		
**D15**	Have you heard of Japanese encephalitis?	Yes ○<br>No ○	 **Go to E1**
**D16**	Can you explain what it is?	Yes: ………. ○<br>No ○	
**D17**	Who can get infected? *Don't read options out loud*	Humans ☐<br>Pigs ☐<br>Other animals: ………. ☐	
**D18**	*If answering "humans":*<br>Do you know how people get infected?	Yes: ………. ○<br>No ○	
**D19**	Has anyone in your family or anyone else you know had Japanese encephalitis?	Yes, someone in the family ☐<br>Yes, someone else ☐<br>No ○	
**D20**	Are you or anyone else in the household vaccinated against Japanese encephalitis?	Yes, the whole family ○<br>Yes, me and/or my partner ○	

		Yes, the children ○<br>No ○	**Go to D22**
**D21**	If yes, who paid for the vaccine?	The family ○<br>The government ○<br>Other: ………. ○	**Go to D23**
**D22**	If no, why not?	Can't afford the vaccine cost □<br>Can't get to the vaccine central □<br>Didn't know there were any available vaccines □<br>Didn't know people could get infected □<br>Afraid of vaccine side effects □<br>Other: ………. □	
**D23**	*If answering "pigs" to qn D17:*<br>Do you know how pigs get infected?	Yes: ………. ○<br>No ○	
No	***E. Disease among the pigs***		
**E1**	Did any of the sows abort their foetuses during the past year?	Yes ○<br>No ○<br>Don't know ○	 **Go to E3**
**E2**	If yes, how many of the sows aborted?	Number: ……….<br>Don't remember ○	
**E3**	Did any of the sows produce stillborn piglets or mummified foetuses during the past year?	Yes, stillborn piglets □<br>Yes, mummified foetuses □<br>No ○	 **Go to E5**
**E4**	If yes, how many sows did it happen to?	Number: ……….<br>Don't remember ○	
**E5**	Have any new-born piglets showed any of the following symptoms during the past year?	Weakness □<br>Shaking □<br>Convulsions □<br>None ○	 **Go to F1**

**E6**	If yes, did any of the piglets die? Approximately how many?	Yes, all of them ○<br>Yes, half of them ○<br>Yes, but only a few ○<br>No, none ○	
No	***F. Mosquito awareness and protection***		
**F1**	Have you heard of diseases being transmitted to people through mosquito bites?	Yes ○<br>No ○	
**F2**	Do you use any mosquito protection in your family?	Yes ○<br>No ○	 **Go to F4**
**F3**	If yes, please describe what kind.	Long-sleeved clothes ☐<br>Mosquito repellents ☐<br>Mosquito nets in windows/doors ☐<br>Mosquito bed nets ☐<br>Mosquito coils ☐<br>Electric rackets ☐<br>Lids on water tanks ☐<br>Other: ………. ☐	**Go to F5**
**F4**	If no, why not?	Can't afford the products ☐<br>Takes too much effort ☐<br>Didn't know you had to protect yourself against mosquito bites ☐<br>Other: ………. ☐	
**F5**	Do you protect your pigs against mosquitos in any way?	Yes ○<br>No ○	 **Go to F7**
**F6**	If Yes, how?	Use mosquito nets ☐<br>Use insect repellents ☐<br>Minimize water sources near the pigs ☐<br>Other: .... ☐	

**F7**	*Observational question:* Is any of the following within sight of the grounds?	Rice field ☐<br>Swamp ☐<br>Lake ☐<br>Pond ☐<br>Well ☐<br>Water tank ☐<br>Other stagnant water: ………. ☐	

Supplementary material 2– Blood sample form

○ Used for questions with only one possible answer

□ Used for questions with more than one possible answer

Farm no: ………. (Example: F1)   Pig no: ………. (Example: P1)

Sex	Age	Breed	*Only sows:* Health history (last 12 months)
Female ○<br>Male ○	…… months<br>*or*<br>…… years	Indigenous ○<br>Exotic ○<br>Mix ○	Abortions □<br>Stillbirths □<br>Mummified foetuses □<br>Weakness, shaking or convulsions in piglets □<br>Number of live piglets in last litter: ………
